# Supplementary material for: Machine learning-aided design and screening of an emergent protein function in synthetic cells
Source: Nat Commun. 2024 Mar 5;15:2010. doi: 10.1038/s41467-024-46203-0 (PMC10914801; doi:10.1038/s41467-024-46203-0)

Supplementary information for

Machine Learning-Aided Design and Screening of an Emergent Protein Function in Synthetic Cells

Shunshi Kohyama†, Béla P. Frohn†, Leon Babl, Petra Schwille*

†These authors contributed equally to this work.

*Corresponding author: schwille@biochem.mpg.de

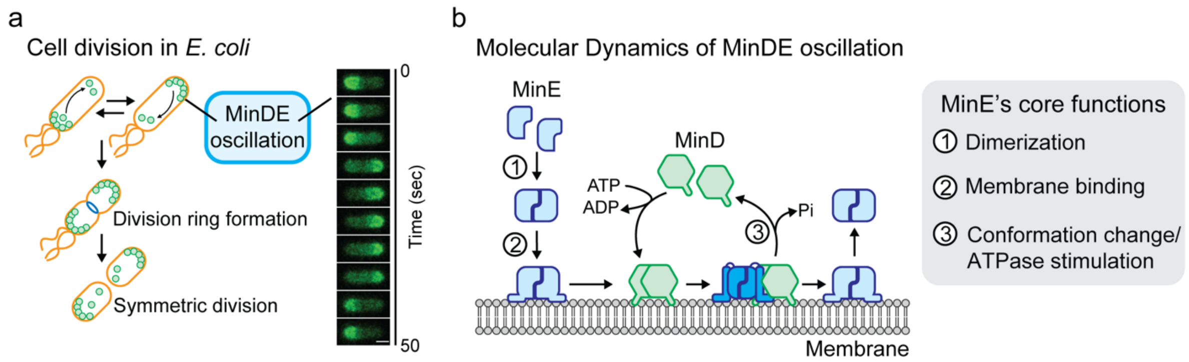

Supplementary Figure 1: Schematic diagram of the MinDE oscillation. a, The oscillatory movement of MinD and MinE proteins determines the division site of *E. coli* cells at the mid-cell region. **b**, Molecular dynamics of the MinDE oscillation. MinD is an ATPase that binds to the membrane upon ADP/ATP exchange. MinE forms a homodimer (1), binds to the membrane (2), and eventually forms the MinDE complex on the membrane (3) together with its conformation change to expose the MinD-interaction helix. Formation of the MinDE complex induces the ATP hydrolysis activity of MinD, and eventually ADP-state MinD and MinE detach from the membrane, forming periodical patterns on the membrane by repeating those processes.

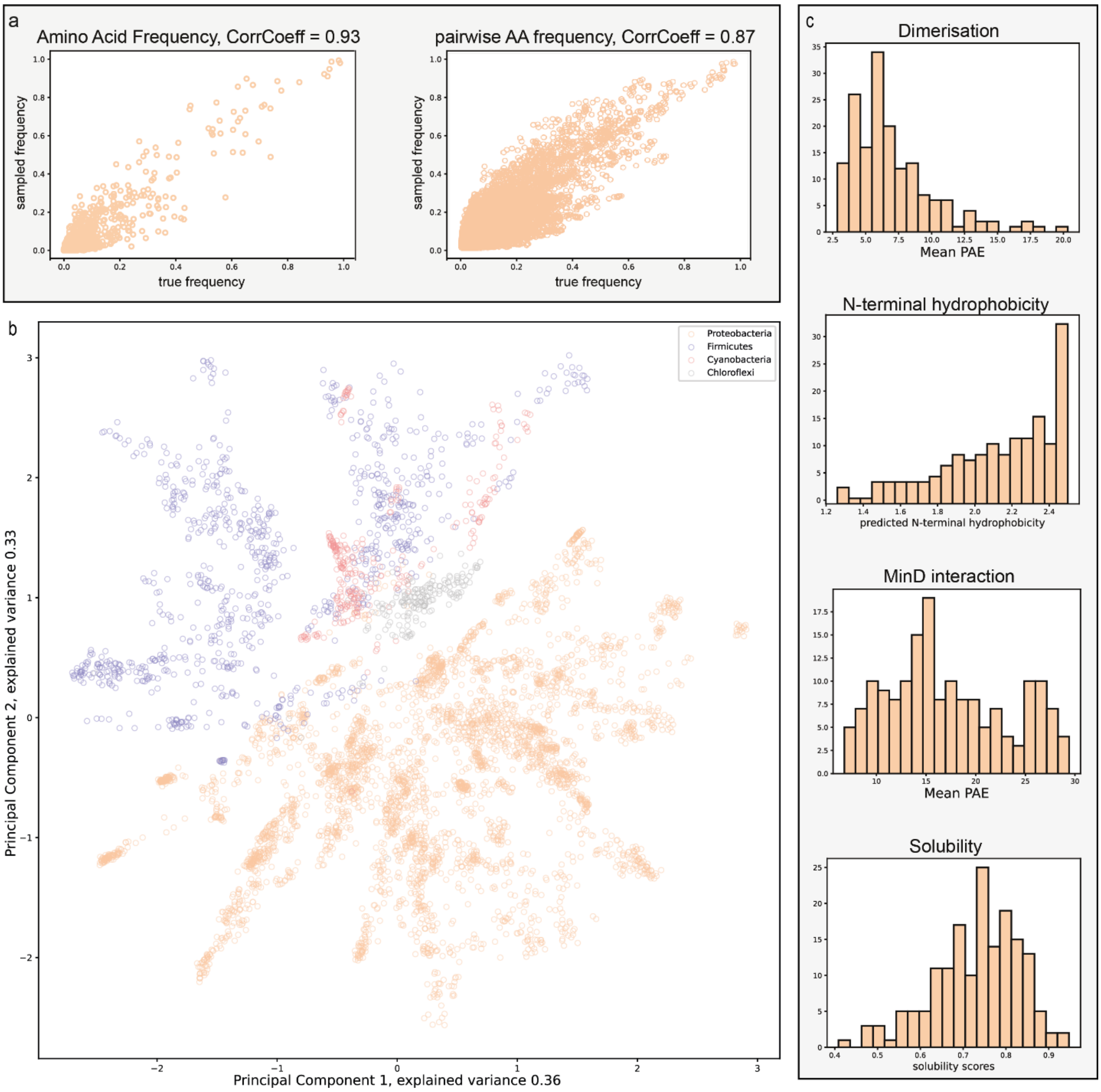

Supplementary Figure 2: Metrics of the protein design and in silico scoring pipeline. a, Evaluation of the VAE. Scatterplot of amino acid frequency and pairwise amino acid frequency in natural MinE sequences (x-axis) and generated MinE sequences (y-axis). **b**, The latent space of the VAE conserves relationships among sequences. Projection of natural MinE variants onto the first two Principal Components of the latent space. Each dot indicates a natural MinE and color indicates phylogenetic group. Only sequences belonging to the four largest phylogenetic groups are displayed. **c**, Histograms of individual scores of generated MinE properties. From top to bottom: (i) Dimerization scores, measured as average Predicted Align Error (PAE) between structured regions of two identical generated MinE proteins. Low scores indicate confidence about MinE dimerization. (ii) Membrane binding scores, measured as average hydrophobicity predicted by ProteinSol Patches[31] at the N-terminal alpha helix. High values indicate high hydrophobicity and hence high probability of membrane binding. (ii) MinD interaction score, measured as average PAE between a generated MinE's MinD-interaction helix and structured regions of MinD. Low scores indicate confidence about MinE-MinD binding. (**iv**) Solubility scores, as calculated by ProteinSol[35]. Values above 0.7 indicate a good predicted solubility.

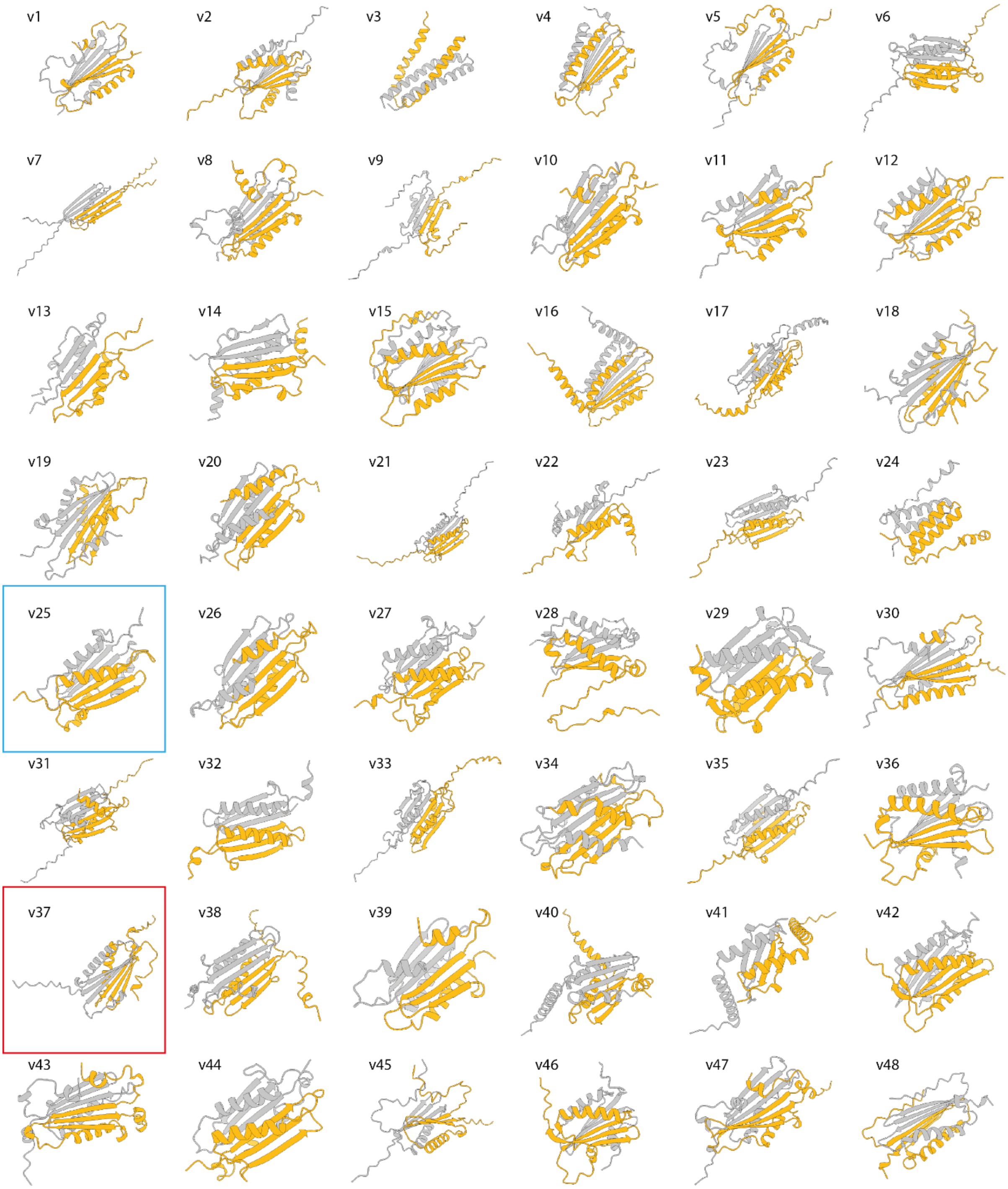

Supplementary Figure 3: Homodimer structures of synMinEv1-48 as predicted by AlphaFold Multimer[30]. The highlighted structures together with Supplementary Figure 4 show that in some low scoring variants no conformational switch is predicted. In the wildtype protein, similar to high-scoring variants (blue outline) the MinE-MinE dimer shows a 3-beta-sheet conformation, which changes to a one-alpha-helix-two-beta-sheet conformation when binding to MinD (Supplementary Figure 4). For some variants, AlphaFold2 did not predict such a change (red outline). Importantly, this occurred in three (synMinEv35, 37, and 48) out of the four variants that had low *in silico* function scores but did show oscillation *in vitro*, indicating that the score was falsely low due to misprediction of AlphaFold2.

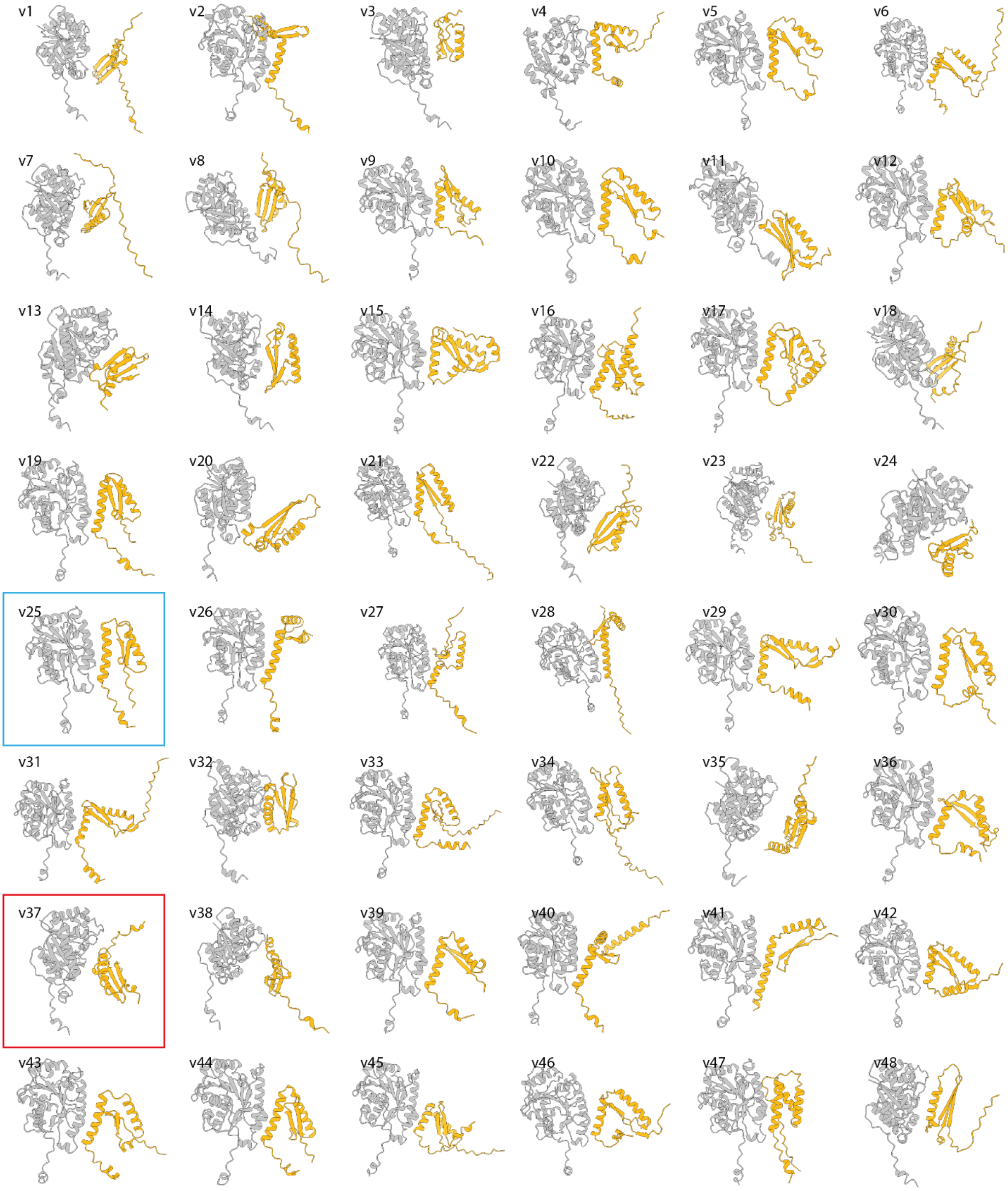

Supplementary Figure 4: Heterodimer structures of synMinEv1-48 as predicted by AlphaFold Multimer[30] . For description of outlines see Supplementary Figure 3.

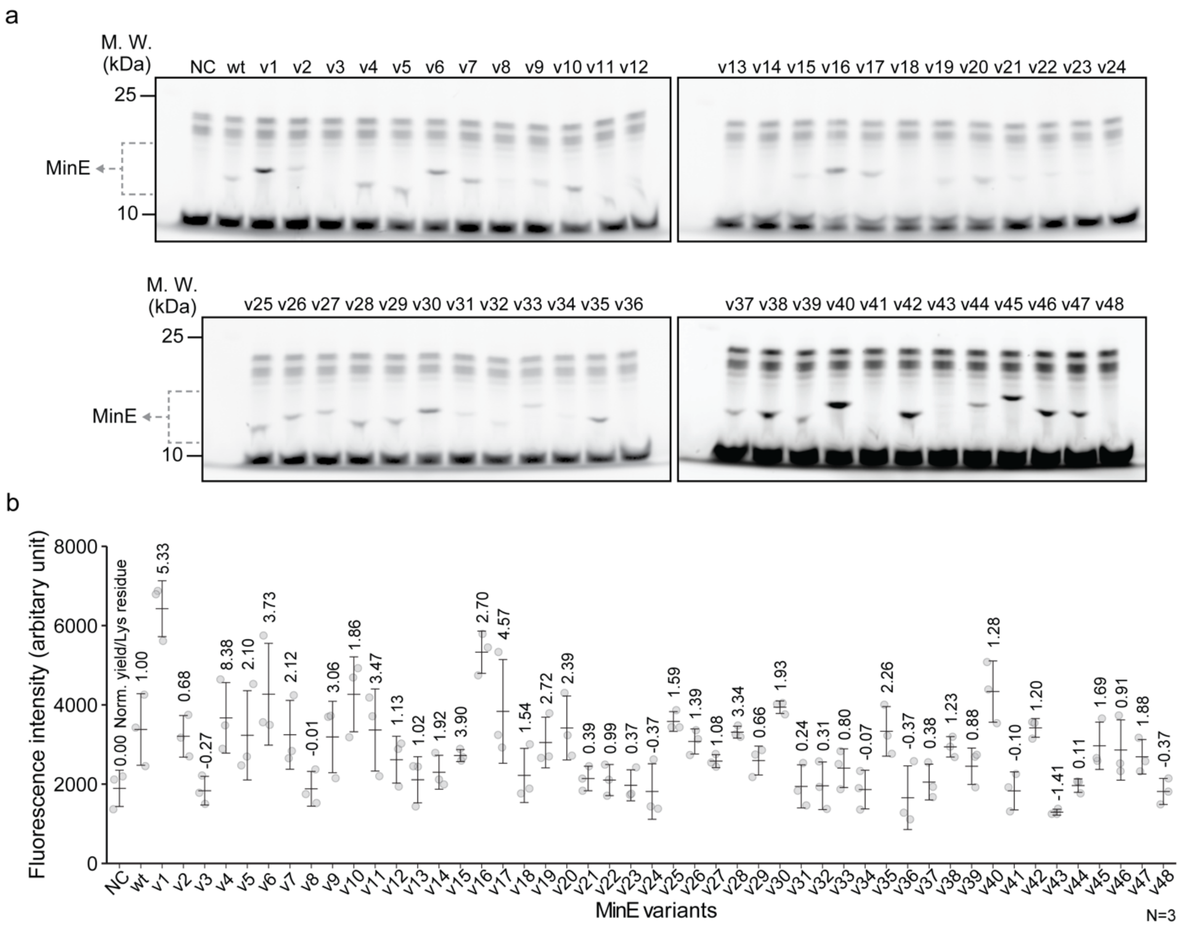

Supplementary Figure 5: *In vitro* expression of synMinE variants. a, All synMinE variants were synthesized in the PURE cell-free expression system and detected by SDS-PAGE using the FluoroTect GreenLys *in vitro* Translation Labeling System. **b**, Estimation of the cell-free expressed yield of synMinE variants confirms that more than 80% (40 variants) of synMinEs were synthesized in the PURE system at detectable levels, while 8 variants (V3, 8, 24, 34, 36, 41, 43, 48) did not get high yield by cell-free expression (Note: However, two of low-yielded variants (v43 and v48) were later positive in the *in vitro* screening (Supplementary Figure 6), indicating such low yield of proteins are still sufficient to induce MinDE dynamics). Plots and bars indicate raw data, average, and standard deviation. N = 3 biologically independent samples are examined over 3 independent experiments. Error bars indicate average ± standard deviation.

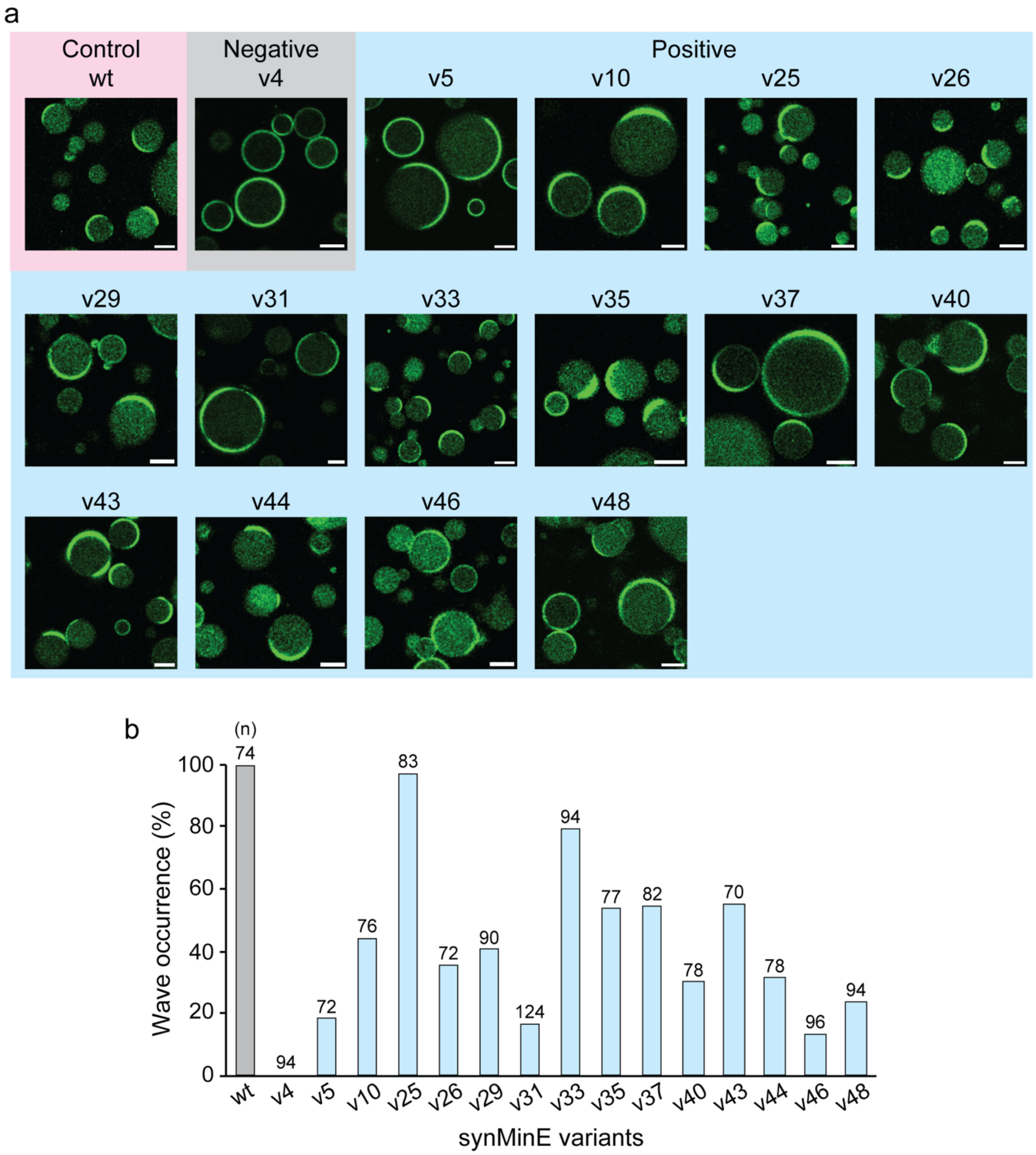

Supplementary Figure 6: *In vitro* screening for functional synMinE variants. a, All synMinE variants synthesized by the cell-free expression system were encapsulated in lipid microdroplets together with MinD and ATP, showing spatiotemporal pattern formation on the membrane with 14 positive variants, while the rest of the negative variants (v4 is shown as a representative example) did not induce dynamic behavior. Later, 10 of the 14 positive variants (v5, 10, 25, 26, 29, 31, 33, 41, 43, 44, and 46) were found to be high-scoring variants from the *in silico* screening. **b**, *In vitro* screening score of positive synMinE variants. synMinEv25 outperformed all the other synMinE variants in wave occurrence and scored close to the wtMinE. The sample sizes (n) are shown above each bar.

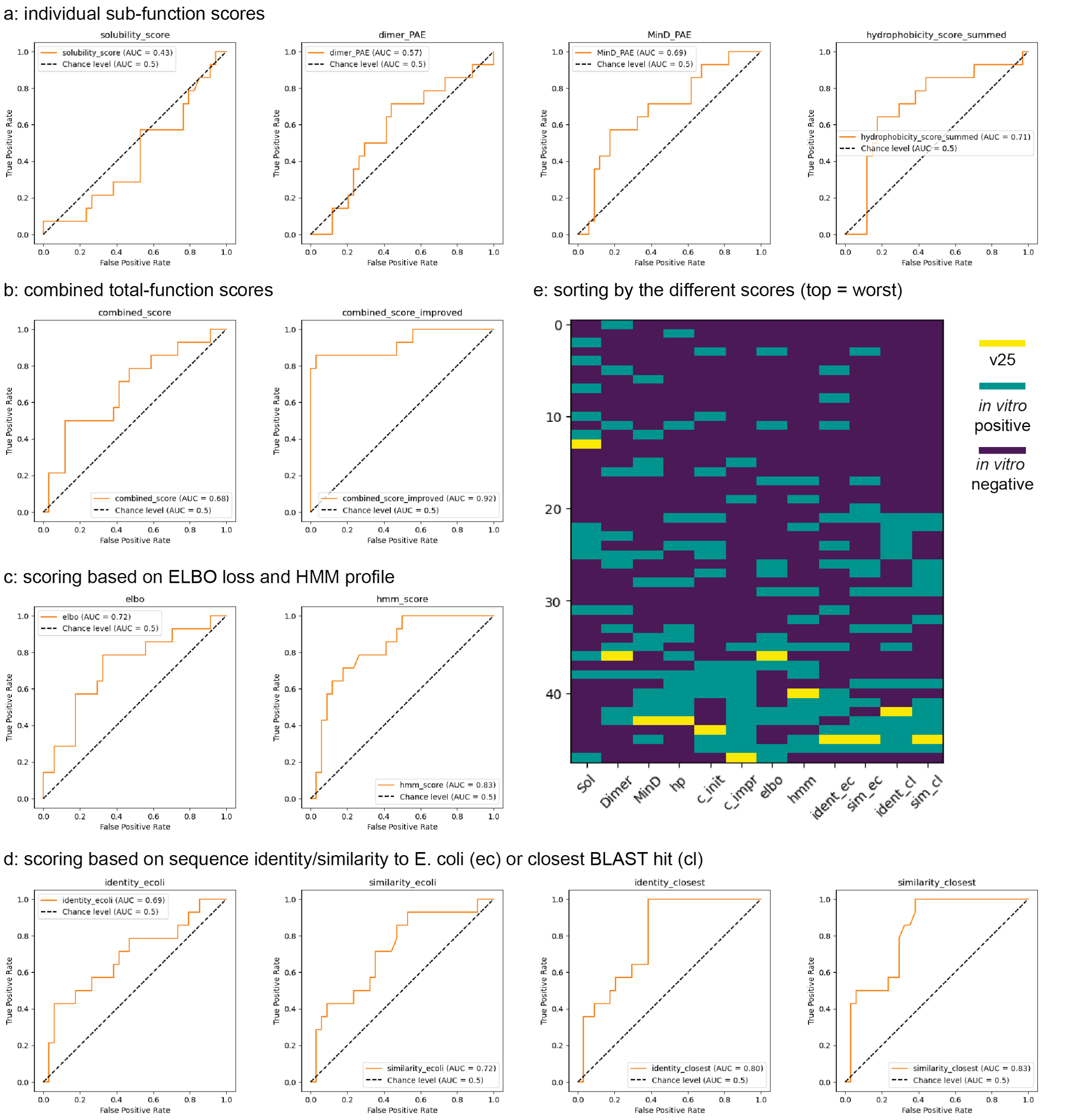

Supplementary Figure 7: Post-Hoc Analysis of *in silico* scoring. After unblinding the sequences, we tested the predictive power of our in silico scoring by generating ROC curves (Receiver Operator Characteristic) and reporting the AUC (Area Under the Curve). **a**, ROC curves for the four sub-function scores individually. The solubility and dimerization scores have only marginal predictive power, whilst the MinD interaction and N-terminal hydrophobicity scores show decent predictive power. **b**, Scores combining the sub-function scores to total-function scores. When combining all four sub-function scores, the overall function score has only limited predictive power (AUC=0.68). However, when combining only the two well-performing sub-function scores (MinD interaction & N-terminal hydrophobicity), a new score, which we call combined_score_improved, the predictive power is immensely increased (AUC=0.92) and importantly outperforms any individual sub-function score. **c**, Scores based on indirect measurement of the VAE likelihood given by the ELBO loss (left) and HMMER scores (right). Importantly, the improved combined score (in **b**) outperforms them both, indicating that a structure-aware "divide-and-conquer" scoring promises to be more effective than scoring by evolutionary conservation or metrics of the generative model. **d**,

Scores based on sequence identity/similarity to *E. coli* (ec) or the closest BLAST hit (cl), where again, the improved combined sore (in **b**) outperforms all those 4 scores, showing the superiority of the "divide-and-conquer" scoring. **e**, bar chart indicating how the 48 tested variants would have been sorted given the 12 different metrics mentioned above. Strikingly, the improved "divide-and-conquer" score ranks synMinEv25 best – the variant which can substitute the wildtype gene fully functionally.

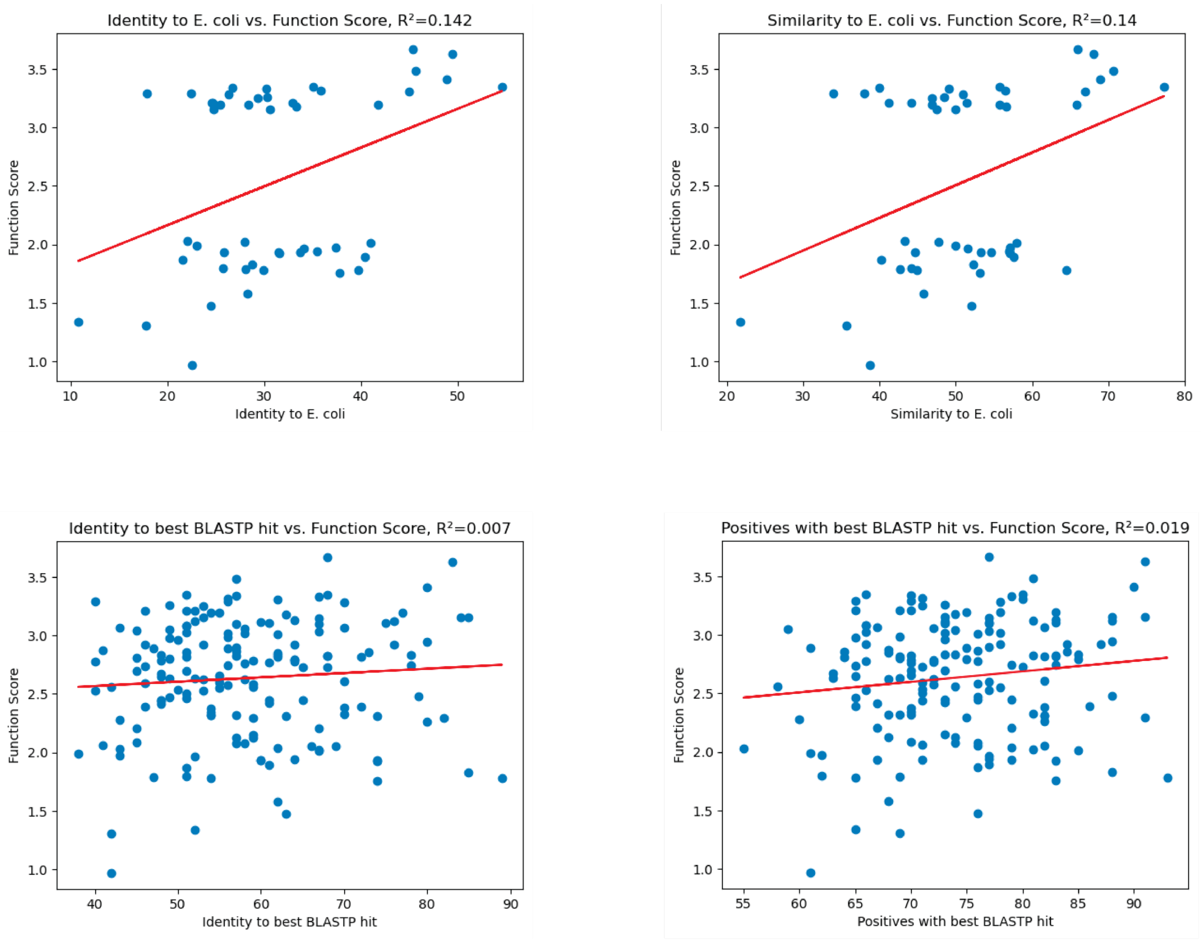

Supplementary Figure 8: Top: Correlation of Function scores of the 48 tested variants vs. sequence identity/similarity to E. coli MinE. Bottom: Correlation of Function scores of all 167 scored sequences vs identity/positives to best BLASTP hit. The low correlations indicate that the function score did not only indirectly measure sequence similarity, but might truly have scored function. BLASTP with default parameters was used to search against the nr database downloaded on October 14 2023.

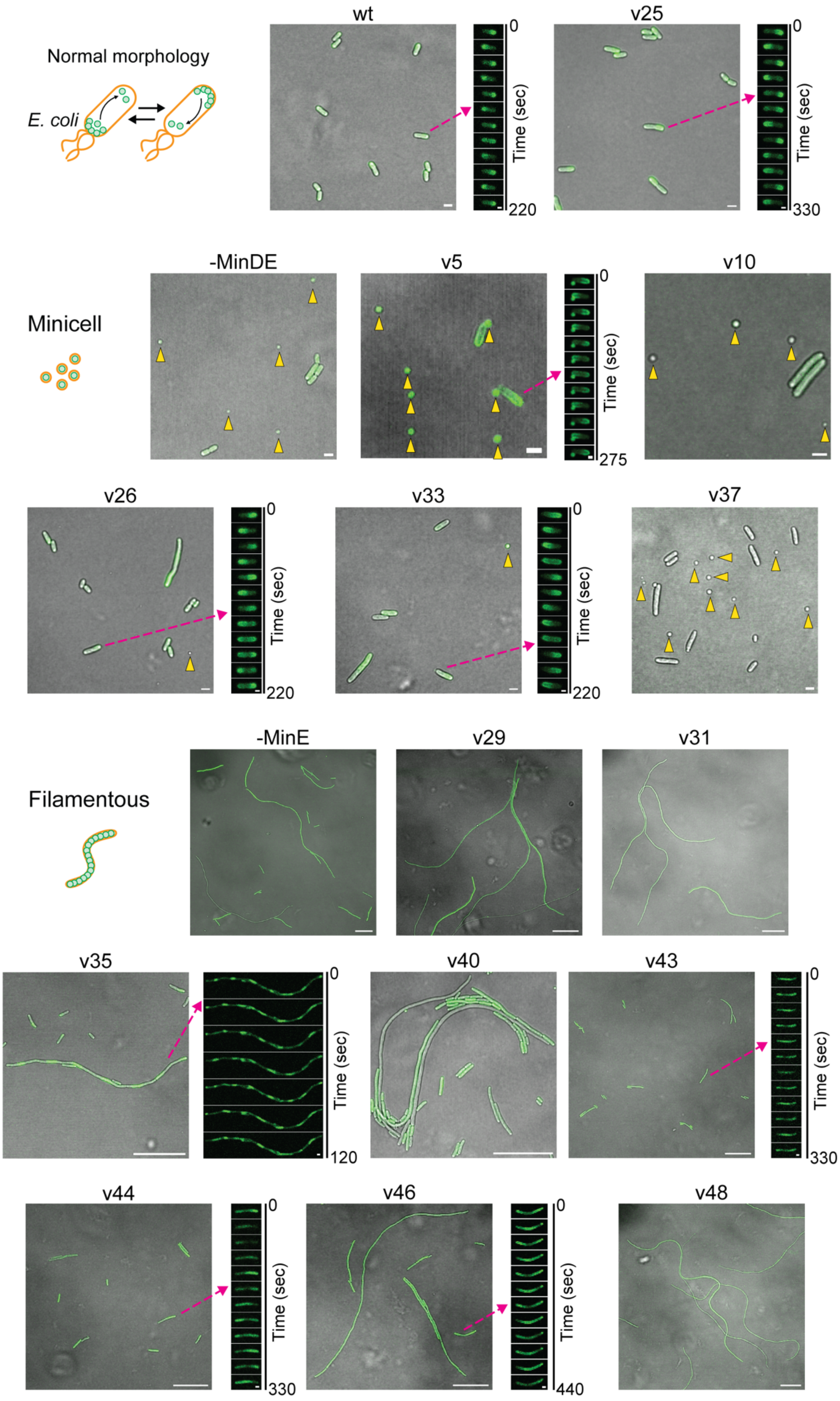
Normal morphology
E. coli
wt
v25
Time (sec)
0
220
330
Minicell
-MinDE
v5
v10
275
v26
v33
v37
Filamentous
-MinE
v29
v31
v35
v40
v43
120
v44
v46
v48
440

Supplementary Figure 9: Morphologies of *E. coli* cells induced by synMinE variants. Wildtype MinE and synMinEv25 indicate normal morphology in Δ*min*DE *E. coli* cells with GFP-tagged MinD, showing that synMinEv25 fully substitutes the wildtype *in vivo*. 5 synMinE variants (v5, v10, v26, v33, v37) together with Δ*min*DE cells (-MinDE) induced minicells (indicated by yellow arrows) due to the lack of proper regulation of cell division, although some variants induced Min oscillations inside the cells. 8 synMinE variants (v29, v31, v35, v40, v43, v44, v46, v48) together with Δ*min*DE cells transformed only with the MinD gene (-MinE) induced filamentous cells due to the inhibition of cell division. However, 4 variants indicate Min oscillations inside the cells, showing MinDE oscillations are partially functional in those conditions. Differential interference contrast and fluorescence images are merged for a wide view of each condition, and fluorescence images are shown for visualizing Min oscillations. Scale bars: 2 µm (normal and minicell) or 20 µm (filamentous) for merged images (wide view) and 1 µm (wt, v25, v5, v26, v33) or 2 µm (v35, v43, v44, v46) for the fluorescence images showing Min oscillations. All the micrographs correspond to a reproducible result from 3 or more independent biological replicates.

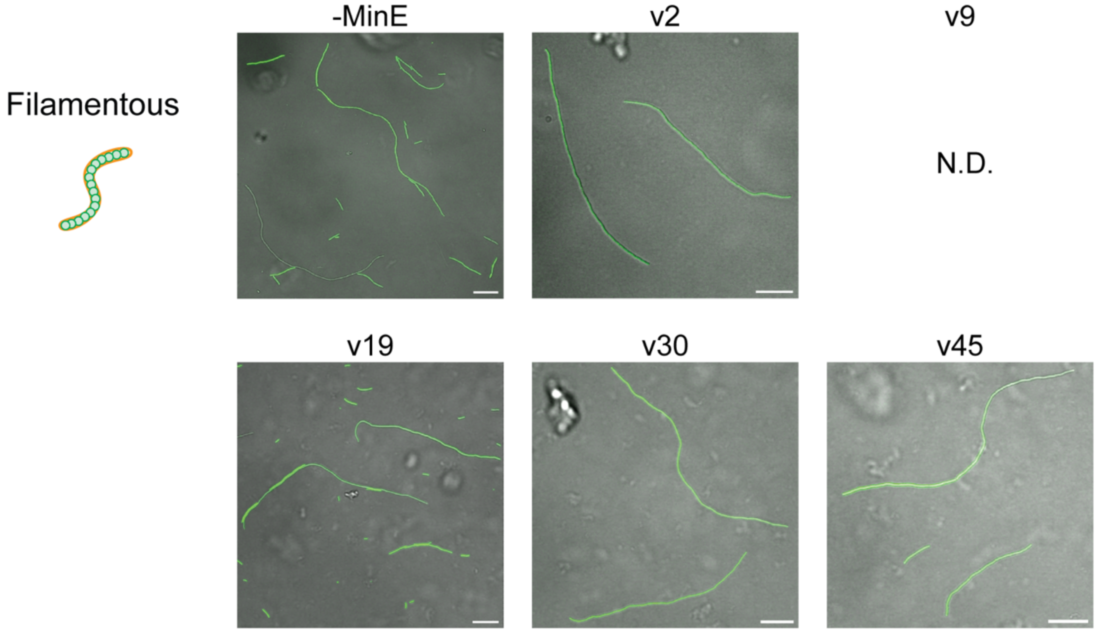

Supplementary Figure 10: Filamentous *E. coli* cells induced by non-functional synMinE variants. The top-5 *in silico* but *in vitro* negative variants (v2, v9, v19, v30, v45) produced filamentous morphology of the cells, and did not induce Min wave dynamics. Note: synMinEv9 could not transform *E. coli* cells with MinD gene after at least 5 bioloical replicates, suggesting this protein is highly toxic to the E. coli cells and therefore results in lethal. Scale bars: 20 µm. All the micrographs correspond to a reproducible result from 3 or more independent biological replicates.

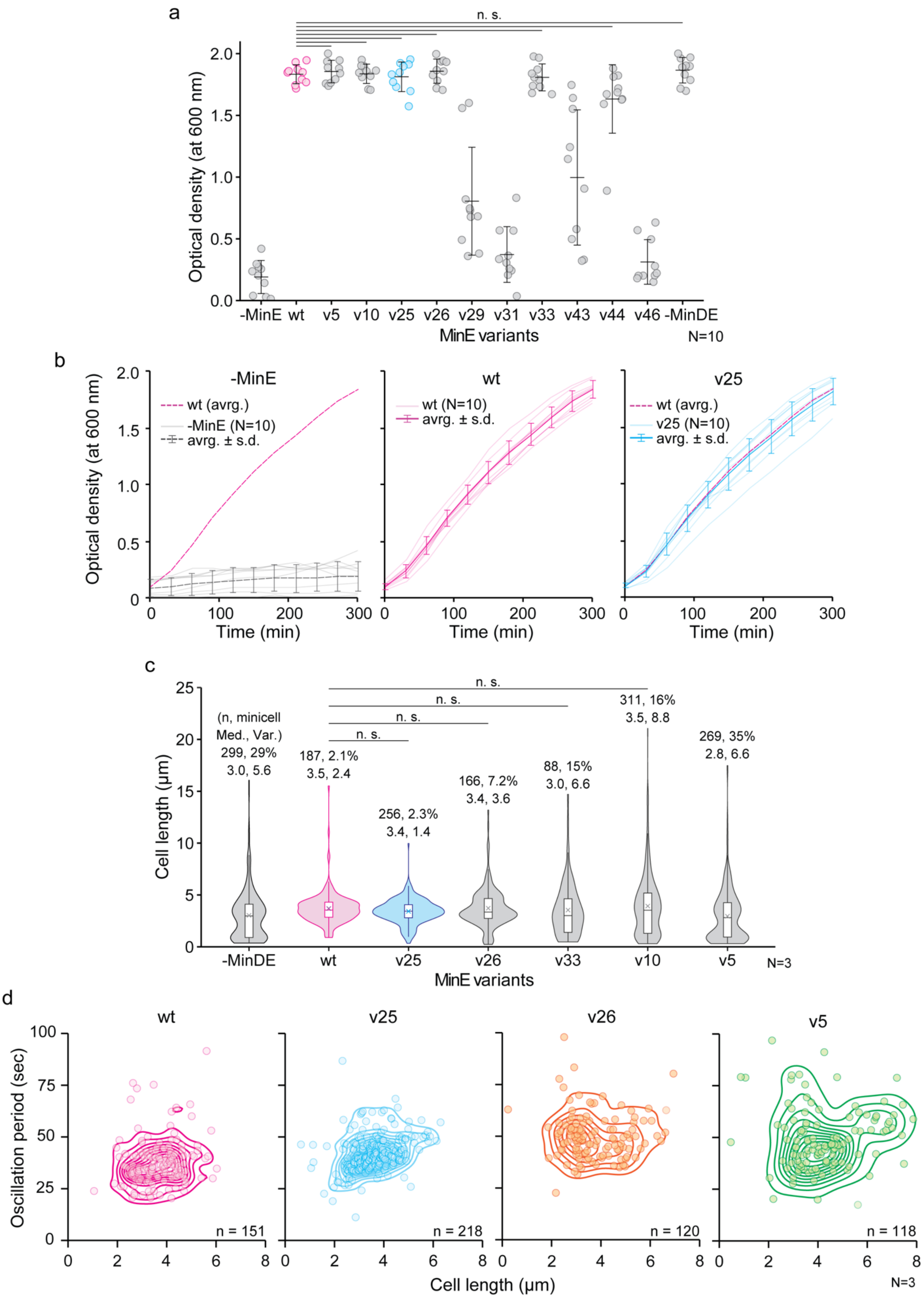

Supplementary Figure 11: Cell growth curve, size, and oscillation period of *E. coli* cells containing synMinE variants. a, Growth of HL1 (Δ*min*DE) cells transformed with synMinE variants together with MinD (OD600 at 300 min of incubation) shows that 6 of 10 variants (v5, 10, 25, 26, 33, and 44) recovered cell growth at the same level as wildtype (n.s. indicates p >

0.05 between wildtype and synMinE variants in double-sided Welch's t-test). Plots and bars indicate raw data, average, and standard deviation. N = 10 biologically independent samples are examined over 10 independent experiments. Error bars indicate average ± standard deviation. **b**, The growth curve of the HL1 cell transformed with wildtype MinE, synMinEv25, or neither of them (shown as -MinE) together with MinD. N = 10 biologically independent samples are examined over 10 independent experiments. Error bars indicate average ± standard deviation. **c**, Violin plots of the size distribution of *E. coli* cells containing synMinE variants. The size distribution of normal and minicell phenotype variants shows that synMinEv25 confers a similar size distribution to wtMinE, while Δ*min*DE (-MinDE) cells and other variants produce higher population of minicells (< 1 µm in cell length). Box plots inside the violin distribution indicate maximum and minimum in 1.5xIQR, 25th and 75th percentile, median (bar), and mean (cross symbol) values. n.s. indicates p > 0.05 in double-sided Mann-Whitney U test. N = 3 biologically independent samples are examined over 3 independent experiments. **d**, Scatter and density plots of oscillation period induced by synMinE variants. Min oscillations induced by wtMinE or three synMinE variants, v5, 25, and v26 show similar period and size distribution in *E. coli* cells, indicating that synMinE variants properly function in *in vivo* environments. N = 3 biologically independent samples are examined over 3 independent experiments.

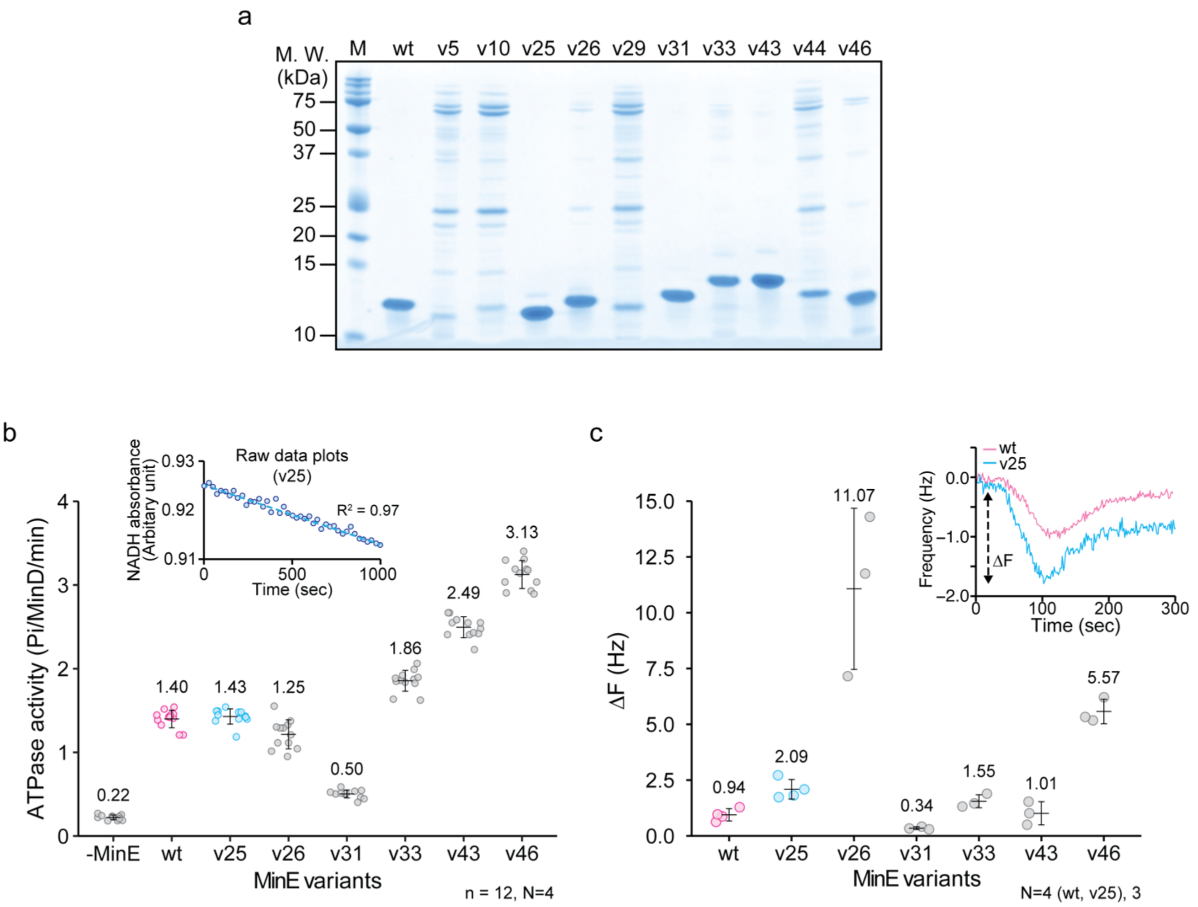

Supplementary Figure 12: Purification of synMinE variants, ATPase activity and QCMD assay. a, 6 of 10 synMinE variants (v25, 26, 31, 33, 43, 46) were obtained from a standard His-tag purification protocol at high yield and therefore used for *in vitro* characterization. **b**, ATPase assays reveal that all tested synMinE variants induce MinD's ATPase activity, although induced ATPase activities vary between 36% (v31) and 226% (v46) compared to wildtype, suggesting ATPase induction has to be finely tuned for proper function of MinDE oscillation. The subset shows the raw data plots of ATPase measurement with synMinEv25. N = 12 biologically independent samples are examined over 4 independent experiments. Error bars indicate average ± standard deviation. **c**, QCMD measurements indicate that all tested synMinE variants bind to the model lipid membrane, although binding strength is highly varying, depending on variants. However, Figure 4d suggests that the difference in membrane binding may not be related to the cell phenotype. The subset shows the comparison of raw QCMD measurement between wt and v25. N = 4 (wt, v25) or 3 biologically independent samples are examined over 4 (wt, v25) or 3 independent experiments. Error bars indicate average ± standard deviation.

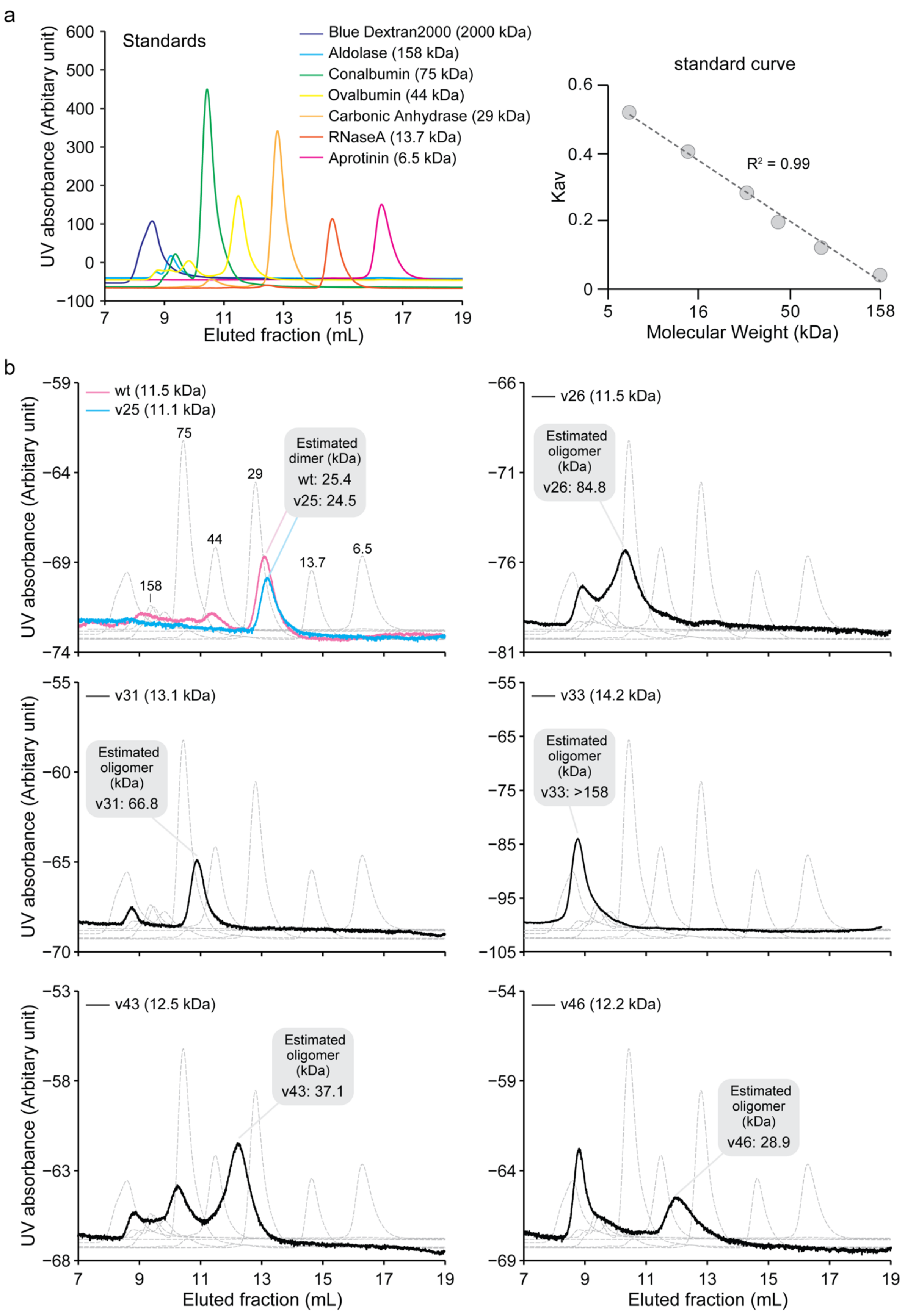

Supplementary Figure 13: Size-exclusion chromatography of synMinE variants. a, Eluted fraction of size-exclusion chromatography for standard proteins and the standard curve. **b**,

Eluted fraction of synMinE variants. synMinEv25 indicates similar eluted peak to the wildtype MinE, showing its proper dimerization. On the other hand, other synMinE variants show distant eluted peaks from wildtype and several peaks within the same variants are also observed, suggesting that oligomers of those variants are bigger than the dimer or tend to be aggregated.

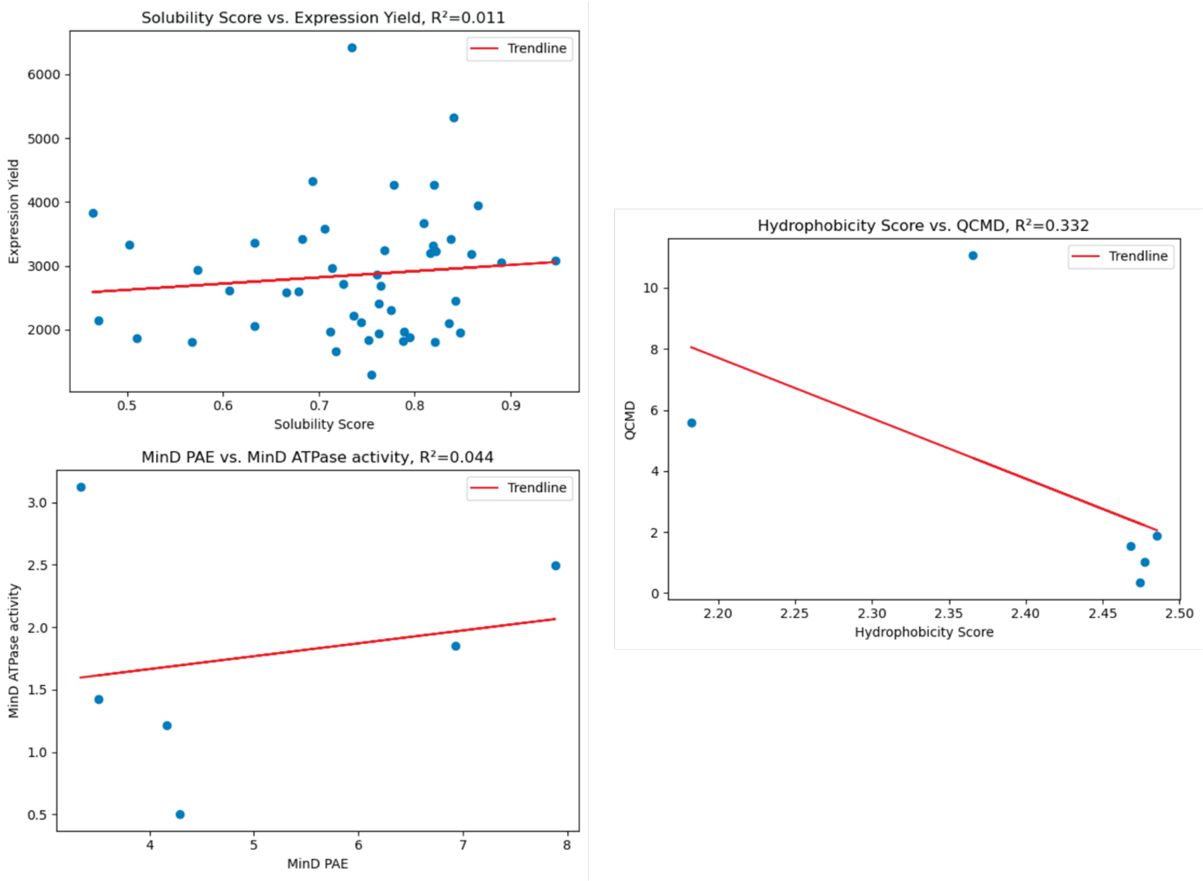

Supplementary Figure 14: Correlation of in silico scores with characteristics of purified proteins. Correlations are minimal, except for the N-terminal hydrophobicity score vs QCMD value, but this should be treated with care as the effect results mostly from two outliers and the sample size is extremely low.

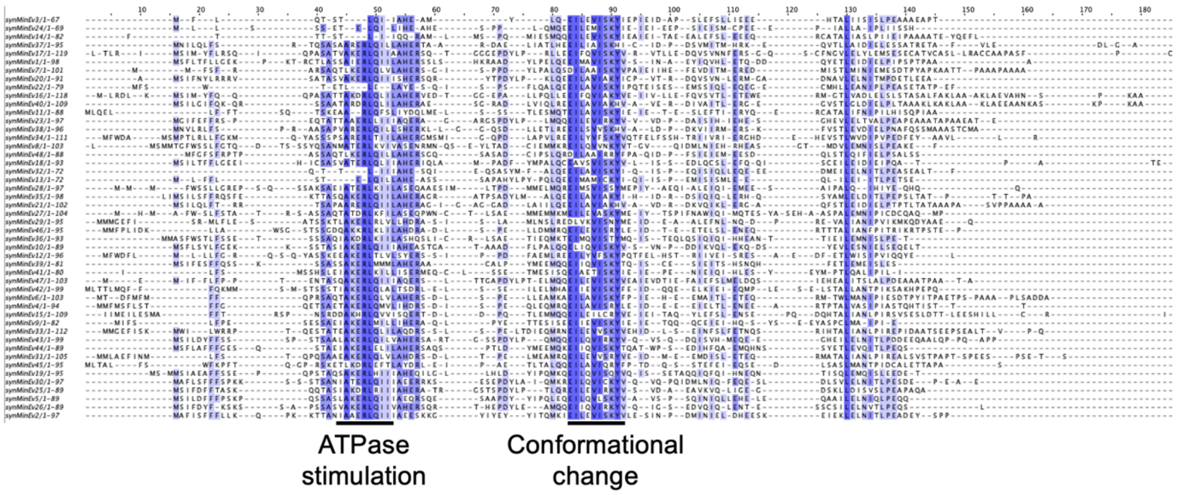

Supplementary Figure 15: Multiple Sequence Alignment (MSA) of all tested variants, as outputted by the MSA-VAE, colored by sequence identity. Regions known to be responsible for specific sub-functions are highlighted [46,49–52]. The MSA is sorted by the function value as used during the *in silico* scoring, from lowest (top) to highest (bottom). Crucial residues are conserved in most sequences, indicating that the MSA-VAE indeed had learned evolutionary constraints. Importantly, several sequences with low scores that did not work *in vitro* show all the crucial residues and regions, indicating that the *in silico* scoring indeed captured some degree of function and only simply sequence similarity.

Supplementary Table 1: DNA sequences and primers used in this study.

Name	Sequence (5’ to 3’)
5’ additional sequence for synMinE library	CCCGCGAAATTAATACGACTCACTATAGGGAGACCACAACGGTTTCCCTCTAGAAATAATTTTGTTTAACTTTAAGAAGGAGATATACCATG
3’ additional sequence for synMinE library	TAACTAGCATAACCCCTTGGGGCCTCTAAACGGGTCTTGAGGGGTTTTTTG
MinD Ins3_FW (for linearization of pMLB-sfGFP-MinD.MinE)	GAATTCGCACGCATTATTGTTG
pMLB-lin-RV (for linearization of pMLB-sfGFP-MinD.MinE)	ATGTATATCTCCTTCTTAAATCTAGA
mGreenLantern-opt-FW (for insertion of mGreenLantern gene)	AGATATACATATGGTTAGTAAAGGAGAAGAAT
MinDuElin-RV (for insertion of mGreenLantern gene)	GAATTCTTTGTAGAGCTCATC
pMLB-ENDlin-FW (for linearization of the pMLB plasmid for synMinE genes)	GCCCGCTGTAAAAGCGCA
MinEdel2-12-RV (for linearization of the pMLB plasmid for synMinE genes)	CATAACTTATCCTCCGA
synMinEv5-OHminD-FW (for insertion of synMinEv5)	AGGATAAGTTATGAGTATTTTAGATTTTTTCTTTCCTTC
synMinEv5-OHpMBL-RV (for insertion of synMinEv5)	TACAGCGGGCTTATTGTTGTTCAGGTAATTGGATG
synMinEv10-OHminD-FW (for insertion of synMinEv10)	AGGATAAGTTATGTCATTTTTATCTTATTTATTTGGTG
synMinEv10-OHpMBL-RV (for insertion of synMinEv10)	TACAGCGGGCTTAAGTAAGTTCCTGCTCAGATAAC
synMinEv25-OH-FW (for insertion of synMinEv25)	AGGATAAGTTATGTCAATTTTTGATTTTTTTACTGC
synMinEv25-OH-RV (for insertion of synMinEv25)	CAGCGGGCTTAGGCTTGCGCGGGAGCCTC
synMinEv26-OHminD-FW (for insertion of synMinEv26)	AGGATAAGTTATGTCAATTTTTGATTATTTTAAATC
synMinEv26-OHpMBL-RV (for insertion of synMinEv26)	TACAGCGGGCTTACAGAGACTGTTCTGGCAG
synMinEv29-OHminD-FW (for insertion of synMinEv29)	AGGATAAGTTATGATGATGGGTGAATTTATTAGTCG
synMinEv29-OHpMBL-RV (for insertion of synMinEv29)	TACAGCGGGCTTACTGTTCGGCCGCGTAATC
synMinEv31-OHminD-FW (for insertion of synMinEv31)	AGGATAAGTTATGATGTTAGCTGAATTTATTAATATG
synMinEv31-OHpMBL-RV (for insertion of synMinEv31)	TACAGCGGGCTTAAGATGTCTCGCTGGGCGA
synMinEv33-OHminD-FW (for insertion of synMinEv33)	AGGATAAGTTATGATGGGTGAATTTATTTCAAAAATG
synMinEv33-OHpMBL-RV (for insertion of synMinEv33)	TACAGCGGGCTTACTGGGGCACAGTTAACGC

Supplementary Table 1: DNA sequences and primers used in this study (continued).

Name	Sequence (5’ to 3’)
synMinEv35-OHminD-FW (for insertion of synMinEv35)	AGGATAAGTTATGTTAATTATGTCAATTTTATCATTTTTTCG
synMinEv35-OHpMBL-RV (for insertion of synMinEv35)	TACAGCGGGCTTATGCCGGTGTTGTTGGCG
synMinEv37-OHminD-FW (for insertion of synMinEv37)	AGGATAAGTTATGAATATTTTACAATTGTTTAGTCGTAC
synMinEv37-OHpMBL-RV (for insertion of synMinEv37)	TACAGCGGGCTTAGGCTCCTAAATCCTCAAGTAC
synMinEv40-OHminD-FW (for insertion of synMinEv40)	AGGATAAGTTATGTCAATTTTAGGAATTTTTC
synMinEv40-OHpMBL-RV (for insertion of synMinEv40)	TACAGCGGGCTTATGCTGCTTTTGGTTTAGACGC
synMinEv43-OHminD-FW (for insertion of synMinEv43)	AGGATAAGTTATGTCAATTTTAGATTATTTCTTTTCAAG
synMinEv43-OHpMBL-RV (for insertion of synMinEv43)	TACAGCGGGCTTACGGCGGGGCTTGTGGTGG
synMinEv44-OHminD-FW (for insertion of synMinEv44)	AGGATAAGTTATGTCATTTTTAGCCTTTTTCTTTG
synMinEv44-OHpMBL-RV (for insertion of synMinEv44)	TACAGCGGGCTTAAGACTGCTCCGGCAGAGT
synMinEv46-OHminD-FW (for insertion of synMinEv46)	AGGATAAGTTATGATGTTTCCATTAATTGATAAATTAT
synMinEv46-OHpMBL-RV (for insertion of synMinEv46)	TACAGCGGGCTTAAGGTTCTGTCGACGGCGT
synMinEv48-OHminD-FW (for insertion of synMinEv48)	AGGATAAGTTATGTTTGGATTTAGTTTTCG
synMinEv48-OHpMBL-RV (for insertion of synMinEv48)	TACAGCGGGCTTAGGAACTCAAAGCGGAGGG
synMinEv2-OHminD-FW (for insertion of synMinEv2)	AGGATAAGTTATGGCATTTATTAGTTTTTTCTTTTTA
synMinEv2-OHpMBL-RV (for insertion of synMinEv2)	TACAGCGGGCTTACGGGGGACTATACTCGTC
synMinEv9-OHminD-FW (for insertion of synMinEv9)	AGGATAAGTTATGATTTTTAGCTTATTTCCTGAATC
synMinEv9-OHpMBL-RV (for insertion of synMinEv9)	TACAGCGGGCTTATTCAATAGGGATGGCCATCAG
synMinEv19-OHminD-FW (for insertion of synMinEv19)	AGGATAAGTTATGTCAATGATGTCAATTGCAGAAG
synMinEv19-OHpMBL-RV (for insertion of synMinEv19)	TACAGCGGGCTTAAGTCTCATCCTCCTCCAAG
synMinEv30-OHminD-FW (for insertion of synMinEv30)	AGGATAAGTTATGGCTTTTTTAAGTTTTTTCTTTTC
synMinEv30-OHpMBL-RV (for insertion of synMinEv30)	TACAGCGGGCTTATTCTGCTTCCGGCTCATCTG
synMinEv45-OHminD-FW (for insertion of synMinEv45)	AGGATAAGTTATGTTAACTGCATTATTTTCATG

Supplementary Table 1: DNA sequences and primers used in this study (continued).

Name	Sequence (5' to 3')
synMinEv45-OHpMBL-RV (for insertion of synMinEv45)	TACAGCGGGCTTAAGATGCGGGCGCCGTTGT
MinD-RV (to obtain pMLB-mGreenLantern-MinD, used with pMLB-ENDlin-FW primer)	TTATCCTCCGAACAAGCG
mGL-opt-Stop-RV (to obtain pMLB-mGreenLantern, used with pMLB-ENDlin-FW primer)	TATTTGTAGAGCTCATCCATGTCATGTG
Linker-His-FW (for linearization of pET28a plasmid)	GGTGGATCTGGAGTCGAGC
del_MinE_rev (for linearization of pET28a plasmid)	CATGGTATATCTCCTTCTTAAAGTTAA
synMinEv5-OHp28a-FW (for insertion of synMinEv5 in pET28a)	GATATACCATGAGTATTTTAGATTTTTTCTTTCCTTC
synMinEv5-OHp28a-RV (for insertion of synMinEv5 in pET28a)	TCCAGATCCACCTTGTTGTTCAGGTAATTGGATG
synMinEv10-OHp28a-FW (for insertion of synMinEv10 in pET28a)	GATATACCATGTCATTTTTATCTTATTTATTTGGTG
synMinEv10-OHp28a-RV (for insertion of synMinEv10 in pET28a)	TCCAGATCCACCAGTAAGTTCCTGCTCAGATAAC
synMinEv25-OHp28a-FW (for insertion of synMinEv25 in pET28a)	GATATACCATGTCAATTTTTGATTTTTTTACTGC
synMinEv25-OHp28a-RV (for insertion of synMinEv25 in pET28a)	TCCAGATCCACCGGCTTGCGCGGGAGCCTC
synMinEv26-OHp28a-FW (for insertion of synMinEv26 in pET28a)	GATATACCATGTCAATTTTTGATTATTTTAAATC
synMinEv26-OHp28a-RV (for insertion of synMinEv26 in pET28a)	TCCAGATCCACCCAGAGACTGTTCTGGCAG
synMinEv29-OHp28a-FW (for insertion of synMinEv29 in pET28a)	GATATACCATGATGATGGGTGAATTTATTAGTCG
synMinEv29-OHp28a-RV (for insertion of synMinEv29 in pET28a)	TCCAGATCCACCCTGTTCGGCCGCGTAATC
synMinEv31-OHp28a-FW (for insertion of synMinEv31 in pET28a)	GATATACCATGATGTTAGCTGAATTTATTAATATG
synMinEv31-OHp28a-RV (for insertion of synMinEv31 in pET28a)	TCCAGATCCACCAGATGTCTCGCTGGGCGA
synMinEv33-OHp28a-FW (for insertion of synMinEv33 in pET28a)	GATATACCATGATGGGTGAATTTATTTCAAAAATG
synMinEv33-OHp28a-RV (for insertion of synMinEv33 in pET28a)	TCCAGATCCACCCTGGGGCACAGTTAACGC
synMinEv43-OHp28a-FW (for insertion of synMinEv43 in pET28a)	GATATACCATGTCAATTTTAGATTATTTCTTTTCAAG
synMinEv43-OHp28a-RV (for insertion of synMinEv43 in pET28a)	TCCAGATCCACCCGGCGGGGCTTGTGGTGG
synMinEv44-OHp28a-FW (for insertion of synMinEv44 in pET28a)	GATATACCATGTCATTTTTAGCCTTTTTCTTTG

Supplementary Table 1: DNA sequences and primers used in this study (continued).

Name	Sequence (5' to 3')
synMinEv44-OHp28a-RV (for insertion of synMinEv44 in pET28a)	TCCAGATCCACCAGACTGCTCCGGCAGAGT
synMinEv46-OHp28a-FW (for insertion of synMinEv46 in pET28a)	GATATACCATGATGTTTCCATTAATTGATAAATTAT
synMinEv46-OHp28a-RV (for insertion of synMinEv46 in pET28a)	TCCAGATCCACCAGGTTCTGTCGACGGCGT

Source Data: Uncropped scan of all blots (fluorescence detection) for estimation of cell-free expression yield of synMinE variants shown in Supplementary Figure 5a

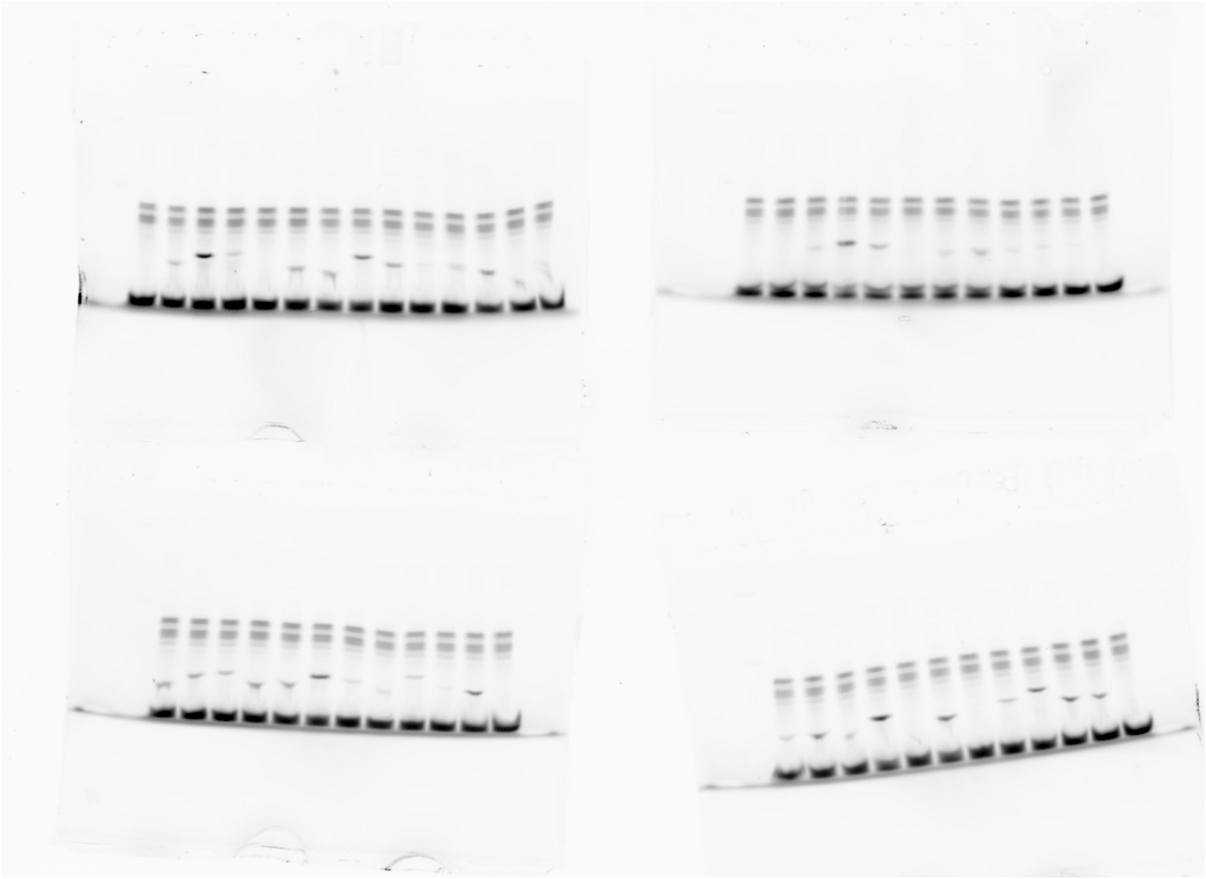

Source Data: Uncropped scan of the gel image (CBB staining) for purification of synMinE variants shown in Supplementary Figure 12a

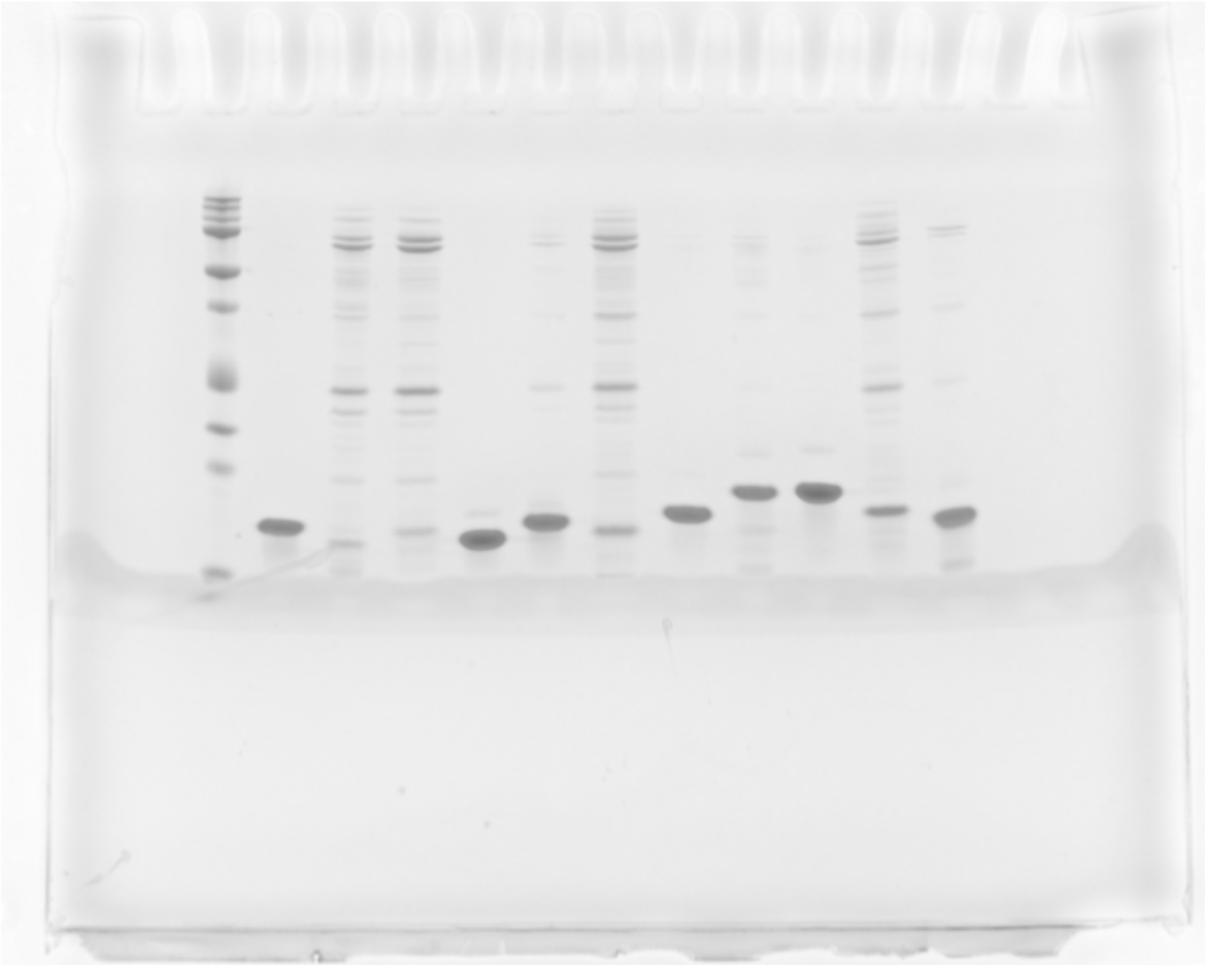

Supplement: Supplementary file 1 — Supplementary Information [file 41467_2024_46203_MOESM1_ESM.pdf]
