# Supplementary material for: Machine learning-aided design and screening of an emergent protein function in synthetic cells
Source: Nat Commun. 2024 Mar 5;15:2010. doi: 10.1038/s41467-024-46203-0 (PMC10914801; doi:10.1038/s41467-024-46203-0)
Supplement: Supplementary file 3 — Description of Additional Supplementary Files [file 41467_2024_46203_MOESM3_ESM.pdf]

Description of Additional Supplementary Files

File Name: Supplementary Data 1
Description: Amino acid and DNA sequences of all synMinE variants used in this study, together with similarity and identity scores to *E. coli* MinE and its closest homolog (with the accession number of homologs). Also, *in silico* ranking, *in vitro* screening results, *in vivo* phenotype, length, and estimated molecular weight are included.

File Name: Supplementary Movie 1
Description: *In vitro* Min oscillations induced by synMinEv25. Min oscillations induced by synMinEv25 inside lipid droplets as a representative "positive" variant found in the *in vitro* screening. Cell-free expressed synMinEv25 was encapsulated in lipid droplets with 1 µM EGFP-MinD, 2.5 mM ATP, and 10 g/L BSA. Then, synMinEv25 self-assembled into spatiotemporal patterns on the membrane together with MinD (shown in green). Timestamp indicates mm:ss. Scale bar: 20 µm.

File Name: Supplementary Movie 2
Description: Inactive Min protein dynamics with a negative synMinE variant. Homogeneous membrane binding of EGFP-MinD (shown in green) together with synMinEv4 inside lipid droplets as a representative "negative" variant found in the *in vitro* screening. Cell-free expressed synMinEv4 was encapsulated in lipid droplets with MinD as mentioned above for synMinEv25, however, they did not induce any dynamics behavior of Min proteins. Timestamp indicates mm:ss. Scale bar: 20 µm.

File Name: Supplementary Movie 3
Description: *In vivo* Min oscillations induced by synMinEv25. Min oscillations inside Δ*min*DE *E. coli* cells transformed with synMinEv25 and mGreenLanternMinD (shown in green). synMinEv25 fulfills the emergent function of Min proteins for cell division and therefore gives normal cell phenotype and their oscillatory movement. Timestamp indicates mm:ss. Scale bar: 10 µm.

File Name: Supplementary Movie 4
Description: Zoomed-in view of *in vivo* Min oscillations. Zoomed-in view of *E. coli* cells showing Min oscillations induced by synMinEv25 and mGreenLantern-MinD (shown in green). Timestamp indicates mm:ss. Scale bar: 2 µm.

File Name: Supplementary Movie 5
Description: Min oscillations in minicell phenotype *E. coli* cells. Min oscillations inside minicell phenotype *E. coli* cells induced by synMinEv5. Minicells can be observed as "spots" together with rod-shaped cells containing Min oscillations (mGreenLantern- MinD is shown in green), indicating synMinEv5 induces Min oscillations but cannot lead to proper cell division. Timestamp indicates mm:ss. Scale bar: 2 µm.

File Name: Supplementary Movie 6

Description: Min oscillations in filamentous phenotype *E. coli* cells. Min oscillations inside filamentous cells transformed with synMinEv37 and mGreenLantern-MinD (shown in green). Even inside the division-defect cells Min oscillations can emerge, hence, some synMinE variants can function as an inducer of Min protein dynamics, but not as a proper cell division machinery. Timestamp indicates mm:ss. Scale bar: 20 µm.

File Name: Supplementary Movie 7
Description: *In vivo* Min oscillations induced by wtMinE. Min oscillations inside Δ*min*DE *E. coli* cells transformed with wtMinE and mGreenLantern-MinD (shown in green) as a positive control. Timestamp indicates mm:ss. Scale bar: 10 µm.
