# Supplementary material for: Machine learning-aided design and screening of an emergent protein function in synthetic cells
Source: Nat Commun. 2024 Mar 5;15:2010. doi: 10.1038/s41467-024-46203-0 (PMC10914801; doi:10.1038/s41467-024-46203-0)
Supplement: Supplementary file 12 — Reporting Summary [file 41467_2024_46203_MOESM12_ESM.pdf]

nature portfolio

Corresponding author(s): Petra Schwille

Last updated by author(s): Feb 6, 2024

Reporting Summary

Nature Portfolio wishes to improve the reproducibility of the work that we publish. This form provides structure for consistency and transparency in reporting. For further information on Nature Portfolio policies, see our Editorial Policies and the Editorial Policy Checklist.

Statistics

For all statistical analyses, confirm that the following items are present in the figure legend, table legend, main text, or Methods section.

n/a	Confirmed	
☐	☒	The exact sample size (*n*) for each experimental group/condition, given as a discrete number and unit of measurement
☐	☒	A statement on whether measurements were taken from distinct samples or whether the same sample was measured repeatedly
☐	☒	The statistical test(s) used AND whether they are one- or two-sided<br>*Only common tests should be described solely by name; describe more complex techniques in the Methods section.*
☒	☐	A description of all covariates tested
☒	☐	A description of any assumptions or corrections, such as tests of normality and adjustment for multiple comparisons
☐	☒	A full description of the statistical parameters including central tendency (e.g. means) or other basic estimates (e.g. regression coefficient) AND variation (e.g. standard deviation) or associated estimates of uncertainty (e.g. confidence intervals)
☐	☒	For null hypothesis testing, the test statistic (e.g. *F*, *t*, *r*) with confidence intervals, effect sizes, degrees of freedom and *P* value noted<br>*Give P values as exact values whenever suitable.*
☒	☐	For Bayesian analysis, information on the choice of priors and Markov chain Monte Carlo settings
☒	☐	For hierarchical and complex designs, identification of the appropriate level for tests and full reporting of outcomes
☒	☐	Estimates of effect sizes (e.g. Cohen's *d*, Pearson's *r*), indicating how they were calculated

Our web collection on statistics for biologists contains articles on many of the points above.

Software and code

Policy information about availability of computer code

Data collection	Python code used to generate novel MinE-like sequences is provided at https://github.com/BelaFrohn/synMinE
Data analysis	Python and R code as well as custom imageJ macros used to analyse data are provided at https://github.com/BelaFrohn/synMinE

For manuscripts utilizing custom algorithms or software that are central to the research but not yet described in published literature, software must be made available to editors and reviewers. We strongly encourage code deposition in a community repository (e.g. GitHub). See the Nature Portfolio guidelines for submitting code & software for further information.

Data

Policy information about availability of data

All manuscripts must include a data availability statement. This statement should provide the following information, where applicable:

- Accession codes, unique identifiers, or web links for publicly available datasets
- A description of any restrictions on data availability
- For clinical datasets or third party data, please ensure that the statement adheres to our policy

All data are available in the text, figures, supplementary information, or source data. Due to their large file size (>10 GB), the original image data files for in vitro screening and in vivo screening are available from the corresponding author upon request. All code is available at https://github.com/BelaFrohn/synMinE . Natural MinE sequences were dowloaded from InterPro on 30/05/2022 and are included in the dataset on GitHub.

Research involving human participants, their data, or biological material

Policy information about studies with human participants or human data. See also policy information about sex, gender (identity/presentation), and sexual orientation and race, ethnicity and racism.

Reporting on sex and gender	n/a
Reporting on race, ethnicity, or other socially relevant groupings	n/a
Population characteristics	n/a
Recruitment	n/a
Ethics oversight	n/a

Note that full information on the approval of the study protocol must also be provided in the manuscript.

Field-specific reporting

Please select the one below that is the best fit for your research. If you are not sure, read the appropriate sections before making your selection.

☒ Life sciences ☐ Behavioural & social sciences ☐ Ecological, evolutionary & environmental sciences

For a reference copy of the document with all sections, see nature.com/documents/nr-reporting-summary-flat.pdf

Life sciences study design

All studies must disclose on these points even when the disclosure is negative.

Sample size	Sample sizes were not predetermined, however, were taken according to the standards of the field (e. g. n>100 for cell phenotype and oscillation characterization and n>10 for ATPase activity (Glock et al., 2018, ACS Synthetic Biology) from at least three independent biological replicates), which ensures sufficient statistics for characterization.
Data exclusions	No data were excluded from the analyses.
Replication	All in vivo screening and in vitro characterization experiment data for statistical analysis were taken from at least three independent biological replicates without any exclusions (frequencies are described in the text). All attempts at replication were successful.
Randomization	Randomization was not applicable in this study since human or animal samples were not analyzed in the study and all experiments were performed under well defined conditions with at least three independent repeats (all attempts at replication were successful).
Blinding	Blinding was applied for the in vitro screening (during analysis of wave/no wave and hence positive/negative result, it was not known which synMinE variant was analysed). Other statistic analyses were only quantitative (e. g. O.D. measurements, size distribution, and oscillation periods) and therefore not applicable to blinding.

Reporting for specific materials, systems and methods

We require information from authors about some types of materials, experimental systems and methods used in many studies. Here, indicate whether each material, system or method listed is relevant to your study. If you are not sure if a list item applies to your research, read the appropriate section before selecting a response.

Materials & experimental systems

n/a	Involved in the study	
☒	☐	Antibodies
☒	☐	Eukaryotic cell lines
☒	☐	Palaeontology and archaeology
☒	☐	Animals and other organisms
☒	☐	Clinical data
☒	☐	Dual use research of concern
☒	☐	Plants

Methods

n/a	Involved in the study	
☒	☐	ChIP-seq
☒	☐	Flow cytometry
☒	☐	MRI-based neuroimaging
